# Rare Loxoscelism-Associated IgG Coombs-Positive Hemolytic Anemia Treated Successfully With Systemic Corticosteroids

**DOI:** 10.7759/cureus.47424

**Published:** 2023-10-21

**Authors:** Anas Alqam, Joud Zakhour, Wissam Karam, Gerson Maldonado, Pavan S Reddy

**Affiliations:** 1 Department of Internal Medicine, University of Kansas School of Medicine-Wichita, Wichita, USA; 2 Department of Hematology and Oncology, University of Kansas School of Medicine-Wichita, Wichita, USA

**Keywords:** immune hemolytic anemia, loxoscelism, brown recluse spider bite, coombs positive hemolytic anemia, warm autoimmune hemolytic anemia

## Abstract

Loxoscelism-associated hemolytic anemia is a rare but critical complication of brown recluse spider bites. It may lead to various systemic manifestations, including jaundice, dark urine, and anemia-related symptoms, in addition to general loxoscelism symptoms such as skin lesions, fever, myalgia, nausea, and vomiting. Prompt diagnosis is crucial and requires recognizing typical laboratory findings such as low hemoglobin, elevated lactate dehydrogenase, reduced haptoglobin levels, and possibly a positive direct antiglobulin test. There is no definitive guideline for the treatment of loxoscelism-associated hemolytic anemia. we report a case of a 32-year-old female who developed severe Coombs-positive autoimmune hemolytic anemia following a brown recluse spider bite, with an improvement in hemoglobin levels and hemolysis indices after the administration of systemic corticosteroids.

## Introduction

Brown recluse spiders are found mainly in the Midwest, South, and West of the United States and rarely outside these endemic areas [1]. The brown recluse (*Loxosceles reclusa*) is prevalent mainly in North America and is a synanthropic spider, meaning its population number increases with the human population. It is mostly encountered within homes in dark, quiet areas (attics, basements, dressers, etc.) [2]. Brown recluse spider bites can lead to various local and systemic complications. Severe hemolytic anemia following such bites is a rare but potentially life-threatening condition known as loxoscelism-associated hemolytic anemia.

Treatment of loxoscelism-associated hemolytic anemia is still unclear. Severe anemia and hemodynamic instability may demand transfusion; plasma exchange and plasmapheresis have been previously used [3,4]. We present a case of a patient who presented with local and systemic manifestations of loxoscelism including Coombs-positive autoimmune hemolytic anemia that was successfully treated with systemic corticosteroids following a brown recluse spider bite.

## Case presentation

A 32-year-old female with an unremarkable medical history presented to the emergency department (ED) with a two-day history of a left shoulder papule that had ruptured, forming a small black lesion, along with subjective fever and body aches following a suspected brown recluse spider bite. At the time of the initial evaluation, she was afebrile but tachycardic. Laboratory tests showed a normal hemoglobin (Hb) level (15.7 gm/dL), normal white blood cell count (7 k/cumm), low platelet count (137 k/cumm), mildly elevated total bilirubin, and aminotransferases (Table 1). She was discharged from the ED after supportive care with a course of oral antibiotics.

**Table 1:** Laboratory investigations at initial presentation

Labs	Results	Reference range
Hemoglobin (Hb)	15.7 gm/dL	12-16 gm/dL
White blood cell (WBC) count	7 k/cumm	5-10 k/cumm
Platelets (PLT)	137 k/cumm,	150-400 k/cumm,
Total bilirubin	2.5 mg/dL	0.0-1.2 mg/dL
​​​​​Aspartate aminotrasferase (AST)	70 U/L	0-32 U/L
Alanine aminotransferase (ALT)	72 U/L	0-32 U/L
Alkaline phosphatase (ALP)	75 U/L	35-104 U/L

**Figure 1:**
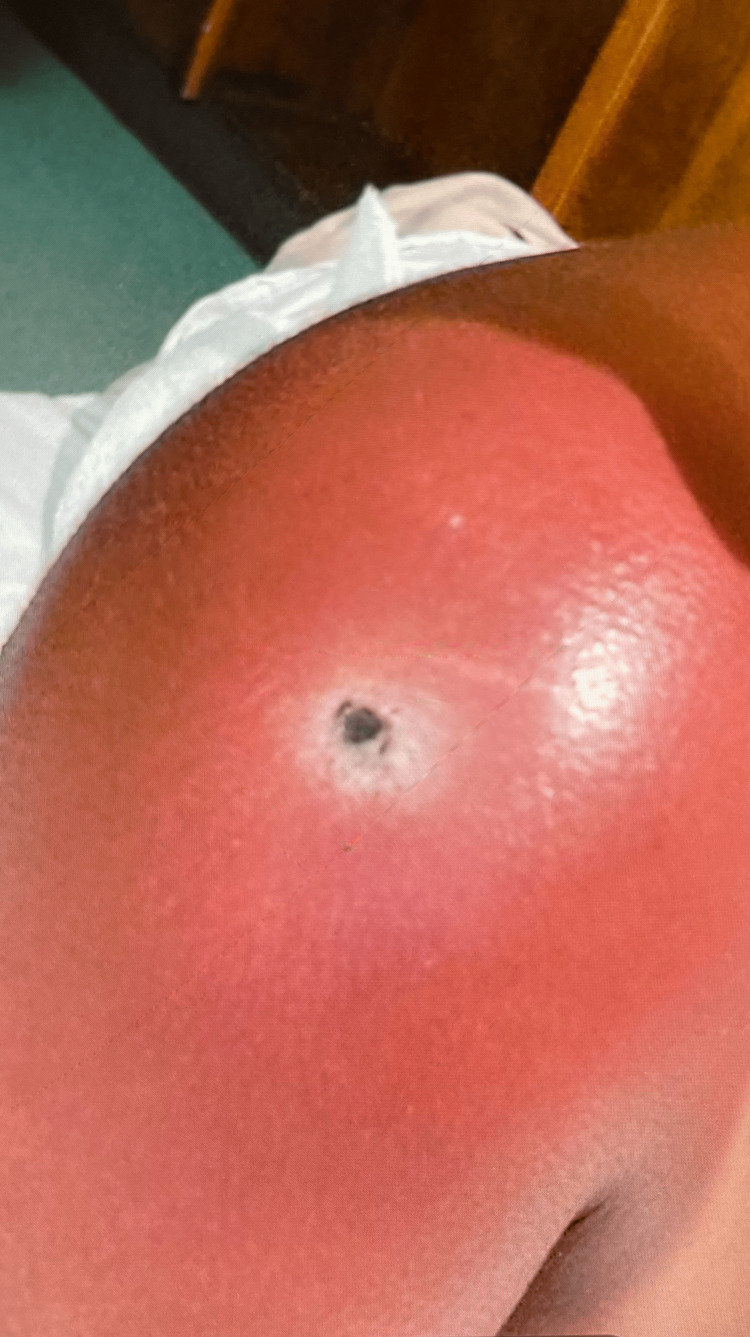
Cutaneous manifestations on the right shoulder upon admission

However, the patient returned to the hospital two days later with progressive erythema and swelling accompanied by pain, nausea, and vomiting. On her second presentation, she was febrile with a temperature of 38.3°C, tachycardic, and blood pressure was stable. Physical examination revealed a sub-centimeter lesion covered by a black eschar, surrounded by erythema extending to the left side of the neck and down to the arm (Figure 1).

Laboratory tests showed a decrease in Hb level (13.2 gm/dL), an increase in total bilirubin (4.4 mg/dL) with elevated unconjugated bilirubin (3.3 mg/dL), and urinalysis revealed +2 blood on dipstick and 0-3 red blood cells per high-power field on microscopy, suggestive of hemoglobinuria (Table 2). Blood cultures were obtained, and the patient was started on intravenous antibiotics for possible cellulitis.

**Table 2:** Laboratory investigations on admission and during hospitalization

Labs	Results	Reference range
Hemoglobin (Hb) on admission and fourth day of hospitalization	13.1 gm/dL, 6.0 gm/dL respectively	12-16 gm/dL
White blood cell (WBC) count	4.9 k/cumm	5-10 k/cumm
Platelets (PLT)	110 k/cumm	150-400 k/cumm
Total bilirubin	4.4 mg/dL	0.0-1.2 mg/dL
Unconjugated bilirubin	3.3 mg/dL	0.0-0.7 mg/dL
Aspartate aminotransferase (AST)	56 U/L	0-32 U/L
Alanine aminotransferase (ALT)	84 U/L	0-32 U/L
Alkaline phosphatase (ALP)	102 U/L	35-104 U/L
Urinalysis	+2 urine blood, RBC 0-3 rbc/hpf	Negative, 0-3 rbc/hpf respectively
Lactate dehydrogenase (LDH)	402 U/L	135-214 U/L
Haptoglobin	< 8 mg/dL	30-200 mg/dL
international normalized ratio (INR)	1.1	0.9-1.1
Prothrombin time (PT)	12.1 sec	9.9-12.9 sec
Activated partial thromboplastin time (aPTT)	28 sec	25-37 sec
D-dimer	955 ng/mL	<500 ng/mL
Fibrinogen	389 mg/dL	200-400 mg/dL

**Figure 2:**
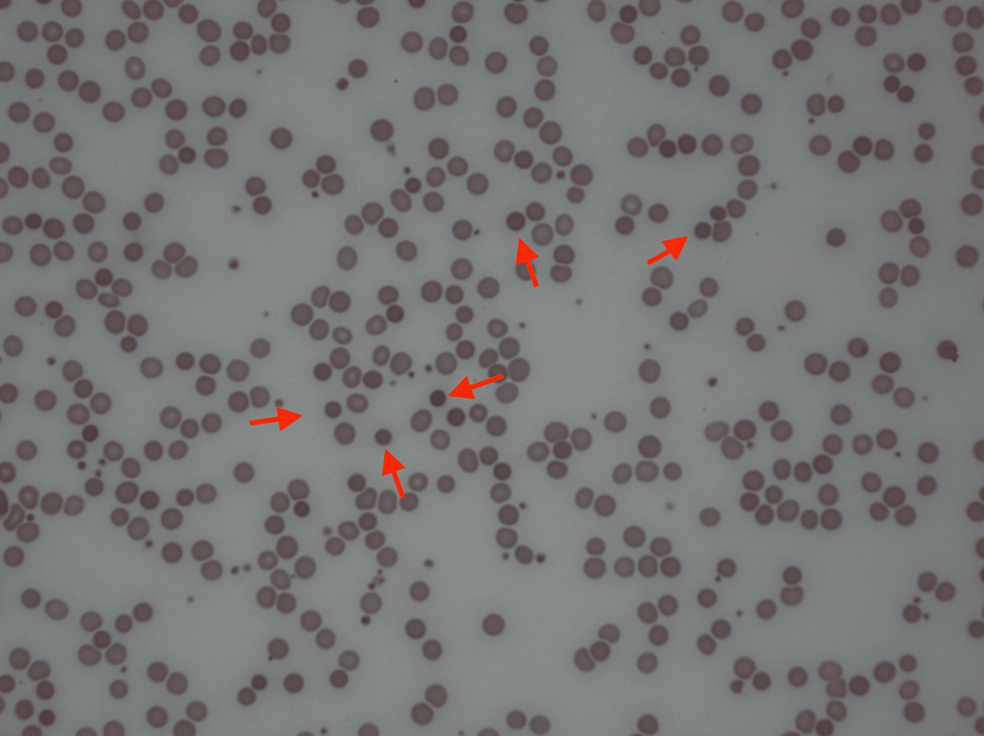
Peripheral blood smear obtained from the patient showing spherocytes (red arrows)

On the fourth day of admission, her hemoglobin level dropped to 6.0 gm/dL. Further workup revealed a low haptoglobin level, elevated lactate dehydrogenase (LDH), and a negative disseminated intravascular coagulation (DIC) panel (Table 2). A peripheral blood smear obtained and reviewed by a pathologist showed severe anemia with spherocytosis (Figure 2).

A polyspecific direct antiglobulin test (DAT) was negative than DAT using monospecific IgG and complement was performed, and the IgG was weakly positive. The hematology team was consulted, and the hemolytic anemia was attributed to loxoscelism. The patient received 2 units of packed red blood cells (PRBCs) and started on prednisone at a dose equivalent to 1mg/kg, along with folic acid supplementation. Her hemoglobin level started to stabilize (8.5 gm/dL), and the patient was discharged to follow up with the hematology-oncology office as an outpatient. At the subsequent follow-up visit two weeks post-discharge, the patient denied anemia-related symptoms, with almost complete resolution of the skin lesion. Repeated lab work revealed elevation of hemoglobin (11.5 gm/dl), normalization of serum LDH, liver enzymes, and total bilirubin (Table 3). 

**Table 3:** Laboratory Investigations on the subsequent follow-up visit post-discharge

Labs	Results	Reference range
Hemoglobin (Hb)	11.5 gm/dL	12-16 gm/dL
Lactate dehydrogenase (LDH)	148 U/L	135-214 U/L
Aspartate aminotransferase (AST)	12 U/L	0-32 U/L
Alanine aminotransferase (ALT)	15 U/L	0-32 U/L
Alkaline phosphatase (ALP)	60 U/L	35-104 U/L
Total bilirubin	0.5	0.0-1.2 mg/dL

## Discussion

The mechanism of hemolysis induced by brown recluse spider bite remains incompletely understood. One theory is that sphingomyelinases in the venom can cause direct RBC hemolysis [5]. There is evidence that *L. reclusa* venom directly attaches to RBC membranes and that RBCs exposed to *L. reclusa* venom failed to undergo hemolysis when exposed to complement-depleted plasma in vitro [6,7]. Gehrie et al. performed an in vitro assay which found that venom naïve erythrocytes had no detectable IgG or complement bound to surface membranes [7]. However, venom sensitization resulted in the deposition of both IgG and complement on the RBC membrane after incubation with ABO-identical fresh frozen plasma. In addition, venom-sensitized erythrocytes but not venom naïve erythrocytes hemolyzed in the presence of ABO-identical fresh frozen plasma. This leads to the belief that complement activation is a major step in the pathogenesis of *L. reclusa* venom-mediated hemolytic anemia. In our case, the combination of low hemoglobin, low haptoglobin, elevated LDH, spherocytes on peripheral blood smear, and IgG-positive Coombs test, all lead to the presumption that RBCs destruction was induced by warm autoimmune hemolytic anemia. 

Warm autoimmune hemolytic anemia is characterized by autoantibodies that target and destroy RBCs, with IgG autoantibodies being the predominant type [8]. These autoantibodies are typically polyclonal panagglutinins directed against common red blood cell antigens, including those in the Rh complex and glycophorin antigens. In most cases, the antibody-antigen complexes are cleared outside the blood vessels by the reticuloendothelial macrophages. However, in severe instances, there can be intravascular hemolysis if the reticuloendothelial system becomes overwhelmed or if complement complexes form on the surface of RBCs.

Approximately 50-60% of warm autoimmune hemolytic anemia cases are associated with an underlying condition. These conditions can include various infections (such as hepatitis C, HIV, Epstein-Barr virus, and babesiosis), lymphoproliferative disorders (like chronic lymphocytic leukemia and multiple myeloma), autoimmune disorders (such as systemic lupus erythematosus, rheumatoid arthritis), and immunodeficiencies (like hematopoietic stem cell transplantation, inherited disorders, solid organ transplant, hypogammaglobulinemia), while the remainder is classified as idiopathic [9-11]. Although rare, there have been reports of both Coombs-positive and Coombs-negative hemolytic anemia in patients due to insect bites, including spider bites [12,13]. In a study by Nguyen N et al. it was observed that out of nine patients who developed moderate to severe loxoscelism, all of them exhibited hemolytic anemia [14]. The average hemoglobin level among these patients was as low as 5.8 g/dL. Interestingly, four out of the nine patients had positive DAT results for both IgG and complement C3 (C3d), while none of them showed positivity for IgG or C3d alone. This is unlike what is observed in our case, in which monospecific DAT was positive for the IgG component only. 

Patients with brown recluse spider bites can often have no recollection of an antecedent bite. They present to medical services after the development of an indurated erythematous rash that could progress to a local black eschar with surrounding desquamation, as well as systemic symptoms such as fever, myalgia, nausea, and vomiting. Our patient had a small, indurated skin lesion followed by rapid progression of an erythematous painful rash and systemic symptoms. Patients should have close observation for progression to life-threatening manifestations associated with loxoscelism such as DIC, rhabdomyolysis, and life-threatening hemolytic anemia. 

Loxoscelism-associated hemolytic anemia may become apparent as early as one day after exposure [15]. In our case, the hemoglobin level was within normal limits on the first encounter with borderline elevated total bilirubin, which might indicate the beginning of the hemolysis process. Elevated total bilirubin and lactate dehydrogenase detect hemolytic anemia before the drop in hemoglobin with a sensitivity of 94% and specificity of 91% [16]. Patients may develop severe symptomatic anemia requiring transfusion support [3]. Other cases reported severe anemia with end-organ damage treated successfully with plasmapheresis or plasma exchange [3,4]. 

In this case, the patient had laboratory findings consistent with the diagnosis of warm autoimmune hemolytic anemia (warm AIHA), therefore, the treatment approach was aligned with the current regimen used for the culprit [10]. First-line treatment includes initiating prednisone with a dose equivalent to 1-2 mg/kg, when the hemoglobin level exceeds 10 and hemolysis indices normalize, which is expected to reach in 70-85% of patients on the second to third week of treatment a slow tapering of the steroids over two to three months can be started [10,17]. There’s supporting evidence that has shown better response rates for rituximab plus glucocorticoids than for glucocorticoids alone in the treatment of warm autoimmune hemolytic anemia [18]. Some experts initiate rituximab if there is no response to corticosteroids after 2-3 weeks. In our case the patient was started on the recommended dose of prednisone and on the subsequent follow-up visit two weeks later, hemolysis indices were within normal limits with improvement in Hb level. 

## Conclusions

A brown recluse spider bite can lead to various local and systemic complications, with severe hemolytic anemia being one of the critical manifestations. Diagnosing loxoscelism-associated hemolytic anemia requires a high index of suspicion, especially in patients with a history of a spider bite and laboratory findings of low hemoglobin, elevated lactate dehydrogenase, low haptoglobin, spherocytes on peripheral blood smear, and a positive direct antiglobulin test. As of now, there are no established definitive treatment guidelines for this condition. In this case, our management aligned with the current strategies used for warm autoimmune hemolytic anemia, resulting in subsequent improvement in hemoglobin levels and hemolysis indices following the initiation of systemic corticosteroids.
